# Platelet-activating factor: an inflammatory mediator in the acute phase of allergic conjunctivitis in a guinea-pig model

**DOI:** 10.1155/S0962935195000317

**Published:** 1995-05

**Authors:** F. Meijer, J. L. van Delft, N. J. van Haeringen

**Affiliations:** 1 Biochemical Laboratory the Netherlands Ophthalmic Research Institute Amsterdam The Netherlands; 2 Department of Ophthalmology Academic Hospital Leiden The Netherlands

## Abstract

The role of platelet-activating factor (PAF) as a mediator of increased conjunctival vascular permeability was investigated in a guinea-pig model of immediate hypersensitivity. Vascular permeability of the conjunctiva was determined by measuring the albumin content in lavage fluid (LF) after topical challenge with either PAF or ovalbumin. PAF produced a dose-dependent increase of the vascular permeability within minutes. Topical pretreatment with levocabastine, a potent histamine H_1_-antagonist demonstrated no effect towards the vascular permeability in response to PAF provocation. Pretreatment with eyedrops containing the specific PAF antagonist BN 52021 (1%) showed a significant inhibition of the vascular permeability (60.2%) and the clinical score (27.5%) after PAF challenge. In sensitized guinea-pigs, levocabastine showed a marked inhibition of both the vascular permeability (80.5%) and the clinical score (70%) after topical challenge with ovalbumin. BN 2021, although to a lesser extent, showed a similar effect towards the vascular permeability (26.8%) and the clinical score (28%) after antigen provocation. When BN 52021 and levocabastine were administered in combination, the vascular permeability was significantly decreased after antigen challenge in comparison with eyes pretreated with levocabastine alone. These results indicate that PAF plays a role in the acute phase of allergic conjunctivitis in the guinea-pig.


Mediators of Inflammation 4, 191–195 (1995)

### Platelet-activating factor: an inflammatory mediator in the acute phase of allergic conjunctivitis in a guinea-pig model

F. Meijer,[1,CA] J. L. van Delft[2] and N. J. van Haeringen[1]

[1]Biochemical Laboratory, the Netherlands Ophthalmic Research Institute, Amsterdam; and [2]Department of Ophthalmology, Academic Hospital, Leiden, the Netherlands

[CA]Corresponding Author

**The role of platelet-activating factor (PAF) as a mediator of increased conjunctival vascular permeability was investigated in a guinea-pig model of immediate hypersensitivity. Vascular permeability of the conjunctiva was determined by measuring the albumin content in lavage fluid (LF) after topical challenge with either PAF or ovalbumin. PAF produced a dose-dependent increase of the vascular permeability within minutes. Topical pretreatment with levocabastine, a potent histamine $H_1$-antagonist demonstrated no effect towards the vascular permeability in response to PAF provocation. Pretreatment with eyedrops containing the specific PAF antagonist BN 52021 (1%) showed a significant inhibition of the vascular permeability (60.2%) and the clinical score (27.5%) after PAF challenge. In sensitized guinea-pigs, levocabastine showed a marked inhibition of both the vascular permeability (80.5%) and the clinical score (70%) after topical challenge with ovalbumin. BN 52021, although to a lesser extent, showed a similar effect towards the vascular permeability (26.8%) and the clinical score (28%) after antigen provocation. When BN 52021 and levocabastine were administered in combination, the vascular permeability was significantly decreased after antigen challenge in comparison with eyes pretreated with levocabastine alone. These results indicate that PAF plays a role in the acute phase of allergic conjunctivitis in the guinea-pig.**

**Key words:** Allergic conjunctivitis, Guinea pig, $H_1$-antagonist, PAF antagonist, Platelet-activating factor (PAF), Vascular permeability

### Introduction

Allergic conjunctivitis is characterized by bilateral hyperaemia, itching, tearing and oedema. Increased vascular permeability has also been described as an important feature of allergic conjunctivitis.[1] It is an immediate hypersensitivity reaction (type I); after interaction of the antigen–IgE antibody complex with the mast cell, the clinical signs and symptoms appear within minutes. Histamine, released from the granules of the mast cell, seems to play a key role but other mediators including leukotrienes (LT), prostaglandins (PG) and platelet-activating factor (PAF) seem to take part in the inflammatory process.[2]

PAF has been shown to induce a wide variety of biological actions such as chemotaxis and activation of neutrophils[3] and eosinophils;[4] moreover PAF induces increased vascular permeability.[5] On a molar basis, PAF appeared to be 1 000 to 10 000 times more potent in inducing vascular permeability changes in skin compared with histamine.[6] Because of these effects, PAF has been implicated in the pathogenesis of allergic diseases including allergic conjunctivitis.

In a number of studies PAF receptor antagonists have been shown to be very effective in inhibiting various effects after PAF challenge in different models. In the eye, PAF antagonists have been shown to be capable of inhibiting corneal oedema formation and pupillary constriction after intracameral injection of PAF[7] and they partially reduced the endotoxin-induced breakdown of the blood–aqueous barrier.[8] Furthermore, the specific PAF antagonist BN 52021 showed a significant inhibition of oedema, leucocyte infiltration and vascularization of the cornea in a rabbit model of immunogenic keratitis[9] after challenge with albumin. In view of these findings, we studied the inhibitory effect of BN 52021 towards microvascular permeability and clinical signs after PAF and antigen provocation in a guinea-pig model of immediate hypersensitivity.

### Materials and Methods

Female Hartley strain guinea-pigs (weight range, 350–450 g) were sensitized to ovalbumin

© 1995 Rapid Communications of Oxford Ltd

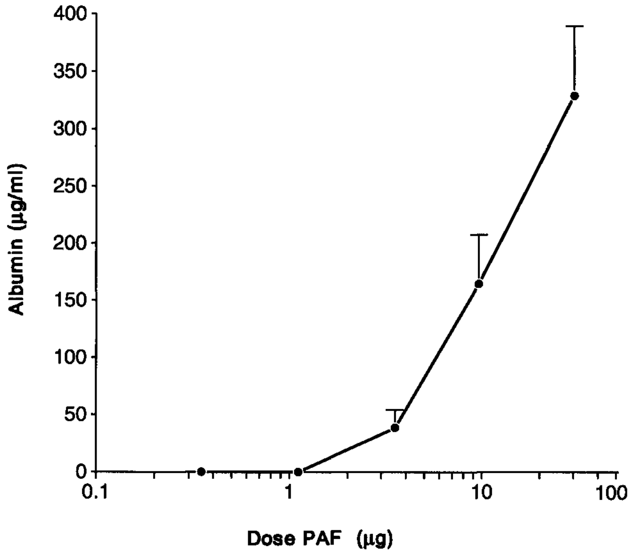

FIG. 1. Dose response curve for PAF on albumin recovery in LF after 30 min. ($n = 3$).

by i.p. injection of a mixture containing 10 µg ovalbumin and 0.1 g aluminium hydroxide suspended in 0.5 ml saline. Two to 3 weeks later, ovalbumin (0.5%) or PAF (10 µg) were applied topically in 25 µl eye drops. Ovalbumin eye drops were prepared as a 0.5% solution in phosphate buffered saline (PBS). Preliminary experiments (not reported here) had shown that this dose of ovalbumin produced a useful response in terms of clinical signs and microvascular permeability.

PAF (C18) was dissolved in water and stored at 4°C according to the manufacturer's instructions. Just prior to the performance of the experiment, the stock solution was further diluted in PBS, using polypropylene tubes and pipette tips.

BN 52021 (1%) or levocabastine (0.05%) were dissolved in 0.3% hypromellose and applied at random to one eye 1 h before challenge with PAF or antigen. The contralateral eye served as a control and received the solvent.

Clinical score (CS) was determined and lavage fluid (LF) was collected at 0.5, 1, 2 and 4 h. LF was sampled by washing the eye with PBS (25 µl) using a polypropylene micropipette, avoiding touching the eye. After three forced blinks the LF was collected and stored at −20°C until used.

Ovalbumin was purchased from Sigma (St Louis, MO), PAF from Cayman (Ann Arbor, MI), guinea-pig albumin anti-serum and guinea-pig albumin from Nordic (Tilburg, the Netherlands). BN 52021 was donated by the institute Henri Beaufour (Le Plessis Robinson, France) and levocabastine hydrochloride was a gift from Janssen pharmaceutics (Beerse, Belgium). Animals were housed and cared in accordance with the guidelines of the ARVO statement for the use of animals in ophthalmic and vision research.

*Clinical score:* For estimation of the inflammatory signs, the total impression of hyperaemia, oedema and swelling was combined in a clinical score (CS) and expressed by visual analogic scales 0–100% by two independent observers.

*Albumin assay:* Total albumin concentration in LF was determined using radial immunodiffusion. Samples were tested in an appropriate dilution. Agar plates (1.5%) containing a 1/100 dilution of guinea-pig albumin anti-serum were used for this purpose. Various concentrations of guinea-pig albumin were used as a standard.

*Statistical evaluation:* Student's *t*-test was used for statistical calculations. A $p$ value $< 0.01$ was taken as significant.

### Results

*Effects of PAF:* Topical application of PAF (10 µg) resulted, within 5 min, in an inflammatory reaction consisting of hyperaemia, conjunctival oedema and periorbital swelling which disappeared within 4 h. The maximum CS was observed after 30 min.

PAF provocation produced a dose related increase of the albumin concentration in LF (Fig. 1). The minimal dose to provoke a leakage of albumin from the conjunctiva vessels was 3 µg/eye. Maximum levels of albumin in LF were found after 30 min, showing a decline to zero 4 h after challenge (Fig. 2). The increase of the vascular permeability with time showed a straight correlation with the development of clinical signs and symptoms.

Pretreatment with BN 52021 showed a significant effect on both the CS (Fig. 3) and the albumin concentration in LF (Fig. 4). The mean albumin content in LF after BN 52021 treatment was inhibited (by 60.2%) in comparison with control eyes.

Levocabastine showed no effect on the CS and failed to reduce the conjunctival microvascular permeability after PAF administration.

*Effects of ovalbumin:* Ovalbumin administration produced a dose-dependent increase in vascular permeability in sensitized animals (data not shown). As a useful concentration for our experiments, a 0.5% solution of ovalbumin was used (dose per eye 125 µg). The inflammatory response to ovalbumin showed a similar clinical reaction in comparison with PAF. After 24 h the

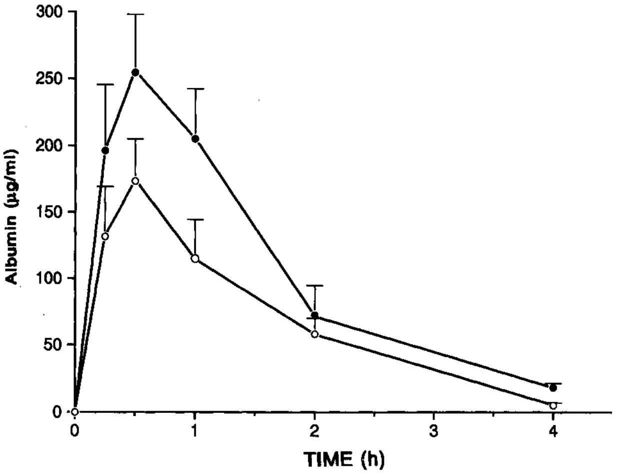

FIG. 2. Time course for albumin recovery in LF in response to PAF (10 µg/eye) and ovalbumin (250 µg/eye) after 30 min ($n$ = 8). PAF, (○); ovalbumin, (●).

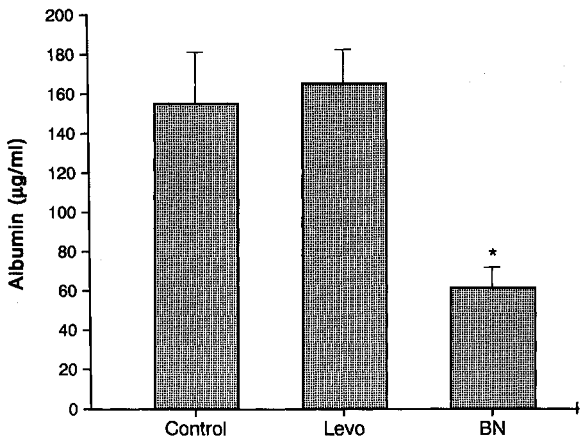

FIG. 4. Effect of levocabastine 0.05% (Levo) and BN 52021 1% (BN) on albumin recovery in LF in response to PAF provocation (10 µg/eye) after 30 min ($n$ = 8).
*Significant difference from eyes receiving only the solvent (control) $p < 0.01$.

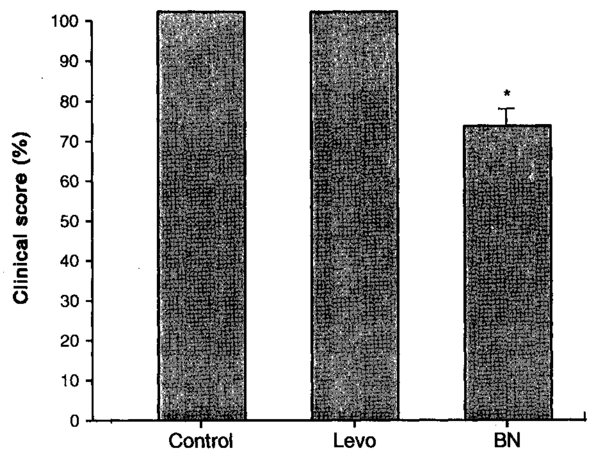

FIG. 3. Effect of levocabastine 0.05% (Levo) and BN 52021 1% (BN) on the clinical score in response to PAF provocation (10 µg/eye) after 30 min ($n$ = 8).
*Significant difference from eyes receiving only the solvent (control) $p < 0.01$.

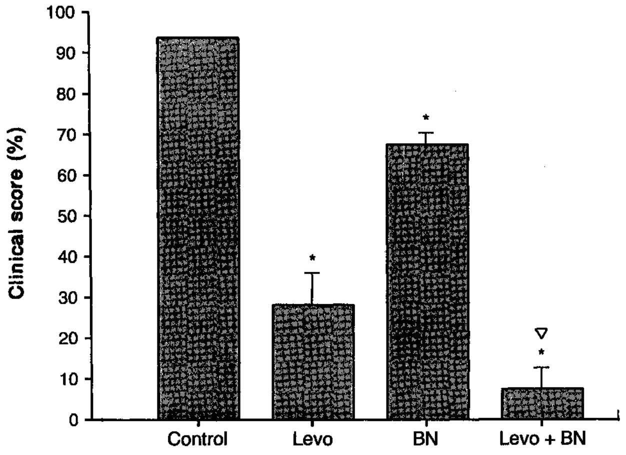

FIG. 5. Effect of levocabastine 0.05% (Levo), BN 52021 1% (BN) and a combination of both (Levo + BN) on the clinical score in response to ovalbumin provocation (125 µg/eye) after 30 min ($n$ = 5–8).
*Significant difference from eyes receiving only the solvent (control) $p < 0.01$
∇Significant difference from eyes receiving levocabastine (Levo) $p < 0.01$.

eyes of the challenged guinea-pigs were free from signs of inflammation.

Levocabastine as a pretreatment significantly reduced the mean of the clinical score (Fig. 5) and showed a significant inhibition (80.5%) of the vascular permeability (Fig. 6). BN 52021 alone showed a significant effect towards the inflammatory response after ovalbumin challenge, reducing the clinical score and inhibiting the vascular permeability (26.8%). The combination of levocabastine (0.05%) and BN 52021 (1%) in one solution showed an even greater inhibitory effect towards the clinical score and vascular permeability (Figs. 5 and 6) in comparison with levocabastine alone. The mixture showed a significant inhibition of the mean clinical score (73%) and the vascular permeability (79%), as compared with the effect of levocabastine alone.

### Discussion

This study shows that PAF plays a role in the acute phase of allergic conjunctivitis. Topical application of PAF to the eyes of guinea-pigs resulted in an inflammatory response including hyperaemia, oedema and swelling of the con-

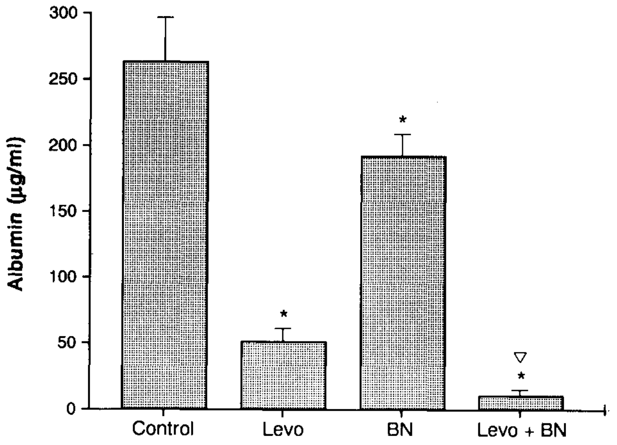

FIG. 6. Effect of levocabastine 0.05% (Levo), BN 52021 1% (BN) and a combination of both (Levo + BN) on the albumin recovery in LF in response to ovalbumin challenge (125 μg/eye) after 30 min ($n = 5-8$).
*Significant difference from eyes receiving only the solvent (control) $p < 0.01$.
▽Significant difference from eyes receiving levocabastine (Levo) $p < 0.01$.

junctiva. Moreover, PAF provocation showed a dose dependent increase in conjunctival vascular permeability. Our results coincide with the observations of others who applied PAF to the eyes of rats[10] and guinea-pigs.[11,12] The dose of PAF needed to produce an inflammatory response of the conjunctiva seems very high when compared with the dose of PAF, less than 0.1 μg, used in other models.[13] In a rabbit model of ocular hypertension, a dose of 250 μg per eye in the presence of 0.25% HSA was topically administered without any systemic side effects.[14] This may be explained by the unique characteristics of the eye towards topical administration of PAF. (Possibly PAF is rapidly converted to lyso-PAF by enzymatic hydrolysis or PAF does not easily penetrate the outer surface of the eye).

Because albumin levels in LF and the CS both reached a maximum after 30 min, we selected this as a time point to test the effectiveness of the specific PAF antagonist BN 52021 and the $H_1$-antagonist levocabastine after provocation with antigen or PAF.

Pretreatment with levocabastine reduced the inflammatory response after topical administration to antigen to sensitized guinea-pigs for the major part (Figs. 5 and 6). Woodward *et al.*[15] observed a similar effect using a combination of $H_1$ and $H_2$-antagonists in guinea-pigs, and in a human study levocabastine appeared to be very effective in relieving the clinical signs and symptoms when used by patients with allergic conjunctivitis.[16] These observations indicate the dominant role of histamine in the acute phase of allergic conjunctivitis. However, a substantial part of the inflammatory response remains present using histamine antagonists as a pretreatment, reflecting the possible involvement of other mediators. Our study shows that pretreatment with BN 52021 alone and in combination with levocabastine before antigen challenge resulted in a significant inhibition of the clinical score and vascular permeability (Figs. 5 and 6). This suggests that the non-histaminergic component of the acute phase of allergic conjunctivitis is at least partly mediated by PAF. Other reports also showed a significant effect of different PAF antagonists on the vascular permeability in various types of inflammation.[8,17]

The mechanism of action of PAF is still not completely understood. PAF receptors situated on target cells are thought to be responsible for the effects of PAF.[18] But it has also been suggested that PAF employs its activity via the secondary synthesis of lipoxygenase products.[19] It is likely that many of the effects attributed to PAF are dependent on the secondary generation of LT and PG. PAF has been shown to release LT,[20] and in a rabbit conjunctival provocation model the inflammatory response after PAF challenge could be inhibited using a lipoxygenase inhibitor.[21] Also, topical administration of LT results in a similar inflammatory response when compared with PAF provocation.[22] However, LT antagonists appeared to be less effective in suppressing the conjunctival vascular permeability after antigen administration in comparison with exogenous LT challenge in guinea-pigs. Because of the significant effect of BN 52021 after ovalbumin challenge, it seems that PAF plays a more prominent role compared to LT in the acute phase of allergic conjunctivitis in guinea-pigs.

Because levocabastine pretreatment showed no effect on CS and vascular permeability after PAF administration, it seems that PAF does not trigger the mast cell to release histamine from its granules. This is confirmed by the observation that during *in vitro* experiments PAF was not able to release histamine from human dispersed cutaneous mast cells.[23]

Because albumin is not produced by the lacrimal gland of the guinea-pig,[24] the concentration of albumin on the ocular surface of the eye is a result of the flow of tissue fluids and other serum proteins across the epithelium to the ocular surface under the influence of several mediators. The LF collected from the surface of the eye at this stage is a mixture of 'leaking' serum and reflex tearing from the lacrimal gland, resulting in some variation between animals but not between the eyes of one animal. Stock *et al.*[11] observed that in the conjunctival system the clinical evaluation was as good as an experimental estimate of the mediator effect measured by extravasation of Evans blue. Because, in our model, clinical score and albumin leakage showed a clear correlation, the observed differences in albumin leakage are due to pharmacological effects and not to the collection method of the LF.

Although the major response in the immediate hypersensitivity reaction seems to be histamine mediated, the effectiveness of BN 52021 in inhibiting the inflammatory response after ovalbumin challenge demonstrates the important role of PAF in the acute phase of allergic conjunctivitis.

### References

1. Tröcme ST. Medical therapy for ocular allergy. *Mayo Clin Proc* 1992; **67**: 557–565.
2. Abelson MB, Schaefer K. Conjunctivitis of allergic origin: immunologic mechanism and current approaches to therapy. *Surv Ophthalmol* 1993; **38**: 115–132.
3. Zoratti EM, Sedgwick JB, Vrtis RR, Busse WW. The effect of platelet-activating factor on the generation of superoxide anion in human eosinophils and neutrophils. *J Allergy Clin Immunol* 1991; **88**: 749–758.
4. Kroegel C, Yukawa T, Dent G, Chanez P, Chung KF, Barnes PJ. Platelet activating factor induces eosinophil peroxidase release from purified eosinophils. *Immunology* 1988; **64**: 559–562.
5. Braquet P, Touqui L, Shen TY, Vergaftig BB. Perspectives in platelet-activating factor research. *Pharmacol Rev* 1987; **39**: 97–145.
6. Hwang SB, Li CL, Lam MH, Shen TY. Characterization of cutaneous vascular permeability induced by platelet-activating factor in guinea pigs and rats and its inhibition by a platelet-activating factor antagonist. *Lab Invest* 1985: **52**: 617–630.
7. Sheng Y, Birkle DL. Intracamerally injected platelet activating factor (PAF) induces marked intraocular inflammatory reactions. *Curr Eye Res* 1992; **11**: 1067–1078.
8. Lin N, Bazan HEP, Braquet P, Bazan NG. Prolonged effect of a new platelet-activating factor antagonist on vascular permeability in an endotoxin model. *Curr Eye Res* 1991; **10**: 1924.
9. Verbeij NLJ, van Haeringen NJ. Interference of a ginkgolide with models of corneal diseases. In: Braquet P, ed. *Ginkgolides — Chemistry, Biology, Pharmacology and Clinical Perspectives.* Barcelona: JR Prous Science Publishers SA, 1988; 749–757.
10. Gautheron PD, Coulbault L, Sugrue MF. A study of PAF-induced ocular inflammation in the rat and its inhibition by the PAF antagonist, L-652731. *J Pharm Pharmacol* 1987; **39**: 857–859.
11. Stock EL, Roth SI, Kim ED, Walsh MK, Thamman R. The effect of PAF, histamine and ethanol on the vascular permeability of the guinea pig conjunctiva. *Invest Ophthalmol Vis Sci* 1990; **31**: 987–992.
12. Woodward DF, Spada CS, Nieves AL, Hawley SB, Williams LS. Platelet-activating factor causes goblet cell depletion in the conjunctiva. *Eur J Pharmacol* 1989; **126**: 23–30.
13. Morley J, Page CP, Paul W. Inflammatory actions of platelet activating factor in guinea pig skin. *Br J Pharmacol* 1983; **80**: 503–509.
14. Jager GV, van Delft JL, van Haeringen NJ, Verbeij NLJ, Braquet P. Antagonist of platelet-activating factor prevents prostaglandin $E_2$ induced ocular hypertension in rabbits. *Prostaglandins* 1993; **45**: 97–105.
15. Woodward DF, Ledgard SE, Nieves AL. Conjunctival immediate hypersensitivity: re-evaluation of histamine involvement in the vasopermeability response. *Invest Ophthalmol Vis Sci* 1986; **27**: 57–63.
16. Abelson MB, George MA, Schaefer K, Smith LM. Evaluation of the new ophthalmic antihistamine, 0.05% levocabastine, in the clinical allergen challenge model of allergic conjunctivitis. *J Allergy Clin Immunol* 1994; **94**: 458–464.
17. Rubin RM, Samples JR, Rosenbaum JT. Prostaglandin-independent inhibition of ocular vascular permeability by a platelet-activating factor antagonist. *Arch Ophthalmol* 1988; **106**: 1116–1120.
18. Evans TW, Chung KF, Rogers DF, Barnes PJ. Effect of platelet-activating factor on airway vascular permeability: possible mechanisms. *J Appl Physiol* 1989; **63**: 479–484.
19. Spencer DA. An update on PAF. *Clin Exp Allergy* 1992; **22**: 521–524.
20. Peplow PV, Mikhailidis D. Platelet-activating factor and its relationship to prostaglandins, leukotrienes and other aspects of arachidonic metabolism. *Prostaglandins Leukot Essent Fatty Acids* 1990; **41**: 71–82.
21. Muller A, Meynier F, Bonne C. PAF induced conjunctivitis in the rabbit is mediated by peptido-leukotrienes. *J Ocul Pharm* 1990; **6**: 227–232.
22. Gary RK, Woodward DF, Nieves AL, Williams SS, Gleason JG, Wasserman MA. Characterization of the conjunctival vasopermeability response to leukotrienes and their involvement in immediate hypersensitivity. *Invest Ophthalmol Vis Sci* 1988; **29**: 119–126.
23. Thomas G, Church MK. Platelet-activating factor does not release histamine from human dispersed cutaneous mast cells. *J Clin Exp Allergy* 1990; **20**: 377–382.
24. Thörig L, van Agtmaal EJ, Glasius E, Tan KL, van Haeringen NJ. Comparison of tears and lacrimal gland fluid in the rabbit and guinea pig. *Curr Eye Res* 1985; **4**: 913–920.

**Received 17 January 1995;**
**accepted in revised form 7 March 1995**
